# Supplementary material for: Late Luteal Subphase Food Craving Is Enhanced in Women with Obesity and Premenstrual Dysphoric Disorder (PMDD)
Source: Nutrients. 2023 Dec 2;15(23):5000. doi: 10.3390/nu15235000 (PMC10707764; doi:10.3390/nu15235000)
Supplement: Supplementary file 1 [file nutrients-15-05000-s001.zip › nutrients-2691201-supplementary/SUPPLEMENTARY FIGURE CAPTIONS.docx]

SUPPLEMENTARY FIGURE CAPTIONS

Supplementary Figure S1. Food Craving Ratings across the Entire Menstrual Cycle.

Supplementary Figure S2. Percent Premenstrual Increase in Symptomatology in PMDD and Healthy Participants.

Supplementary Figure S3. QQ Plot of the Unadjusted Linear Mixed Model.
