# Supplementary figures and images for: Late Luteal Subphase Food Craving Is Enhanced in Women with Obesity and Premenstrual Dysphoric Disorder (PMDD)
Source: Nutrients. 2023 Dec 2;15(23):5000. doi: 10.3390/nu15235000 (PMC10707764; doi:10.3390/nu15235000)

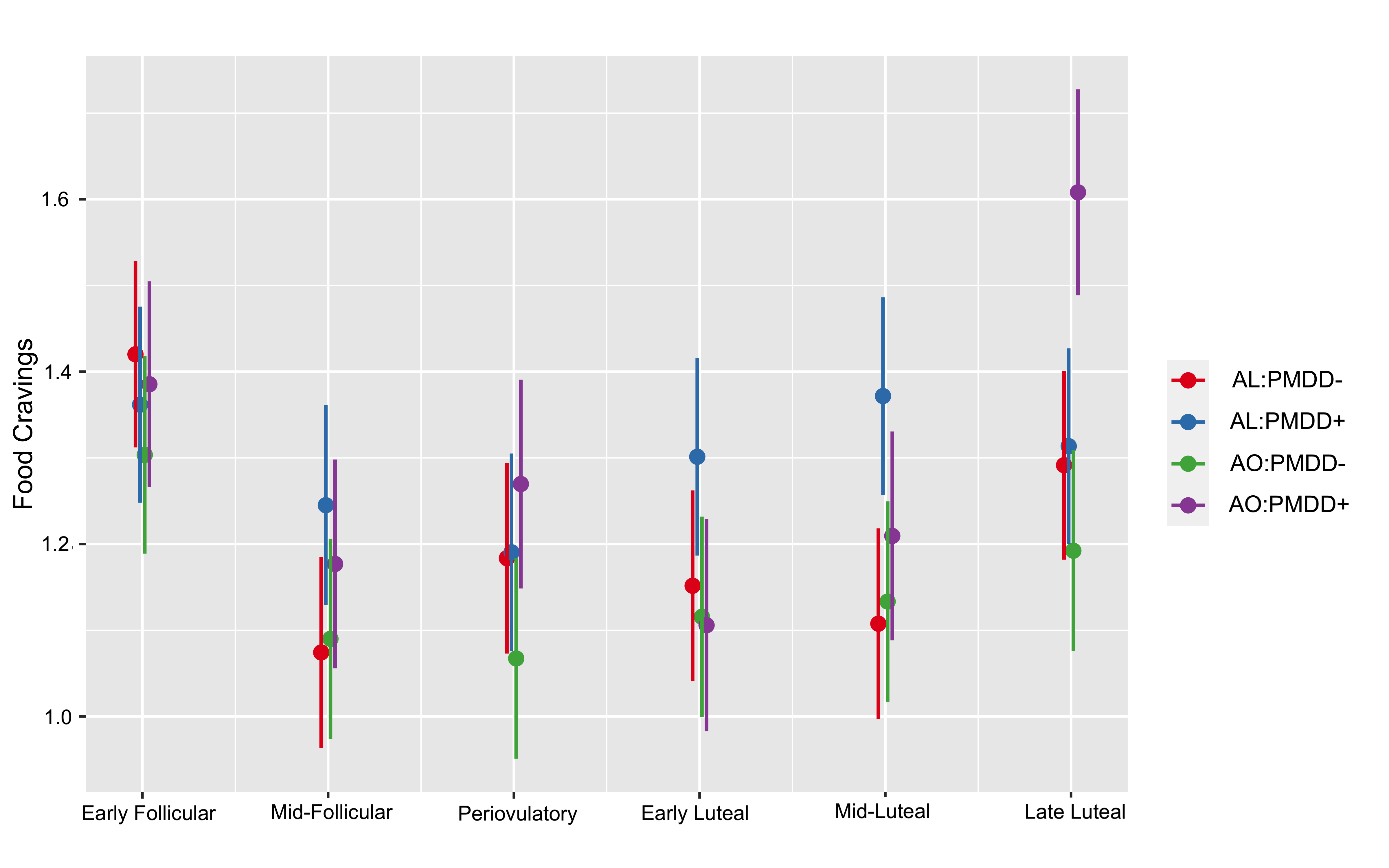

Supplement: Supplementary file 1 [file nutrients-15-05000-s001.zip › nutrients-2691201-supplementary/Supplementary.Figure S1.png]

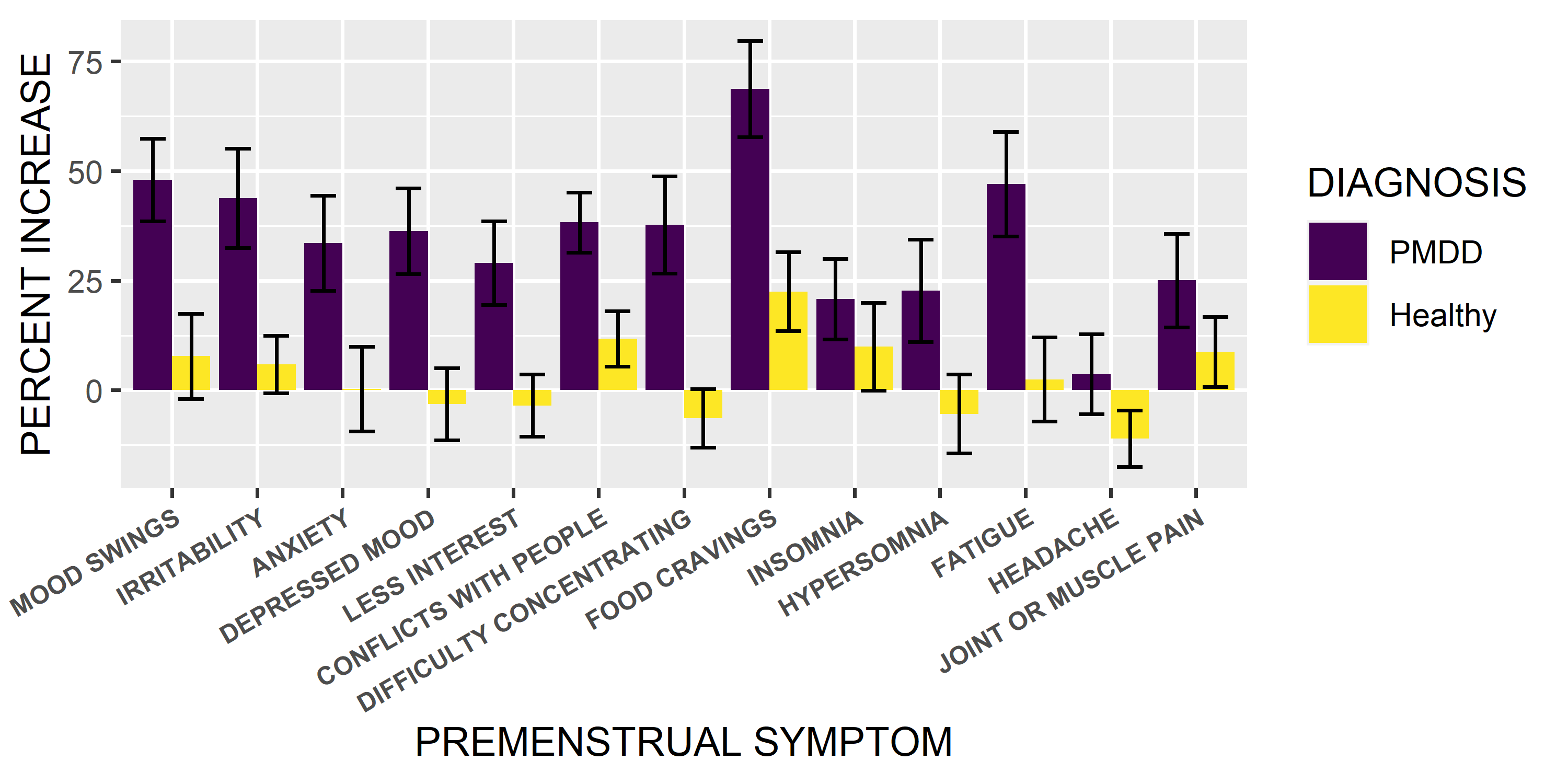

Supplement: Supplementary file 1 [file nutrients-15-05000-s001.zip › nutrients-2691201-supplementary/Supplementary.Figure S2.tiff]

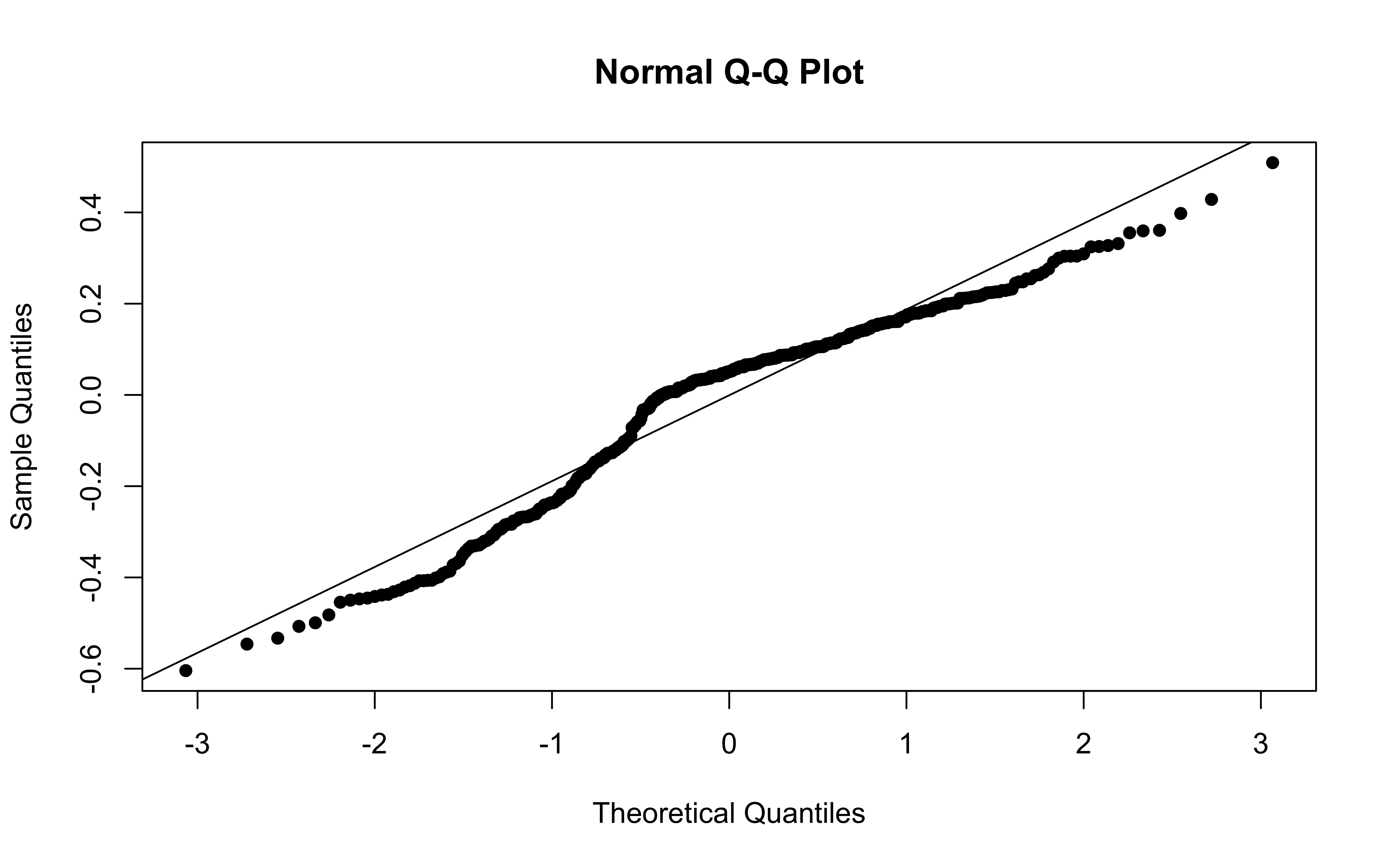

Supplement: Supplementary file 1 [file nutrients-15-05000-s001.zip › nutrients-2691201-supplementary/Supplementary.Figure S3.png]
